# Monitoring Animal Behaviour and Environmental Interactions Using Wireless Sensor Networks, GPS Collars and Satellite Remote Sensing

**DOI:** 10.3390/s90503586

**Published:** 2009-05-13

**Authors:** Rebecca N. Handcock, Dave L. Swain, Greg J. Bishop-Hurley, Kym P. Patison, Tim Wark, Philip Valencia, Peter Corke, Christopher J. O'Neill

**Affiliations:** 1 Commonwealth Scientific and Industrial Research Organisation (CSIRO), Livestock Industries, Private Bag 5, Floreat, WA, 6014, Australia; 2 CSIRO, Livestock Industries, JM Rendel Laboratory, Ibis Avenue, North Rockhampton, QLD, 4701, Australia; E-Mails: Dave.Swain@csiro.au (D.L.S.); Greg.Bishop-Hurley@csiro.au (G.J.B-H.); Kym.Patison@csiro.au (K.P.P.); Christopher.O'Neill@csiro.au (C.J.O.); 3 CSIRO, ICT Centre, P.O. Box 883, Kenmore, QLD, 4069, Australia; E-Mails: Tim.Wark@csiro.au (T.W.); Philip.Valencia@csiro.au (P.V.); Peter.Corke@csiro.au (P.C.)

**Keywords:** animal-landscape interactions, cattle, social behaviour, high fix-rate GPS, telemetry data, extensive cattle system

## Abstract

Remote monitoring of animal behaviour in the environment can assist in managing both the animal and its environmental impact. GPS collars which record animal locations with high temporal frequency allow researchers to monitor both animal behaviour and interactions with the environment. These ground-based sensors can be combined with remotely-sensed satellite images to understand animal-landscape interactions. The key to combining these technologies is communication methods such as wireless sensor networks (WSNs). We explore this concept using a case-study from an extensive cattle enterprise in northern Australia and demonstrate the potential for combining GPS collars and satellite images in a WSN to monitor behavioural preferences and social behaviour of cattle.

## 1. Introduction

Reducing the environmental impact of animals can be assisted by monitoring their behaviour and correlating it with environmental information to determine optimal management intervention strategies [1,2]. However, monitoring is complicated by the need to record animal movement concurrently with landscape condition, which in itself influences the animals' behaviour [3]. There is a long history of ecologists and environmental scientists using radio-transceivers and position data from the Global Positioning System (GPS) to track and monitor the behavioural ecology of free ranging animals [4-7]. This increasing availability of technologies for the remote collection of telemetry data and the widespread use of satellite-based earth-observation has led to researchers combining these technologies to help them understand animal behavioural responses [8], although the full integration of these technologies is still under development. More recently there has been a focus on combining data from different sensing platforms using emerging technologies such as wireless sensor networks (WSNs) which enable a broad range of information to be transmitted wirelessly and facilitate analysis of the data collected by the devices worn by the animals [9]. This new generation of WSNs presents both challenges and opportunities for monitoring animal behaviour and their interaction with the environment.

We define a wireless sensor as a device that measures a physical quantity and can transmit this information wirelessly to another location. Wireless sensor networks are typically comprised of a collection of sensors with their own power supply, wireless communication, data storage, and data processing capability. Using communications between sensor nodes, data from any node can be channelled back to the gateway node and then to the internet. Networks of embedded devices that work together to provide enhanced monitoring across spatial and temporal scales are growing in popularity [10]. Optimizing the performance of WSNs is the focus of ongoing computer science based research [9]. Wireless sensor networks are increasingly being used in terrestrial monitoring applications by ecologists and environmental scientists to collect and transmit data from remote field sites back to base [11,12]. The majority of current WSN deployments utilise sensors at fixed locations [11,13] where each node typically contains multiple sensors to measure a number of environmental parameters, for example, soil moisture or micro-climate. There are also some recent examples of WSN nodes being fitted to animals, creating a collection of mobile nodes within a WSN [9,14]. Within natural extensive environments communication within such networks of mobile nodes creates a unique set of challenges [15] which will be discussed in this paper.

In fragile landscapes domesticated livestock pose a risk to the environment through overuse of particular areas [16-18]. Overgrazing areas of the landscape by herbivores can reduce plant diversity and ground cover, with associated risks of increased erosion [19,20]. Monitoring landscape condition is a prerequisite to implementing appropriate animal management strategies. In extensive grazing environments monitoring landscape condition using traditional observation methods is difficult and costly, as is the management of animals over large extents. Multi-spectral remotely-sensed images can be used to map the temporal changes in rangeland condition [21]. However, multi-spectral images from satellite-based sensors only provide an indirect measurement of physical characteristics and their usefulness is realised through the interpretation and calibration of the image data.

There are many methods for interpreting remotely-sensed images (see [22] and [23] for good overviews); qualitative methods which combine spatial and spectral analysis include identifying spatial patterns in the image data, the presence of low- or high-regions, and changes in size or shape of the patches in classified maps. Useful qualitative information can also be calculated from image data which, depending on the landscape characteristic being studied, ranges from simple vegetation indices such as the widely used Normalized Difference Vegetation Index (NDVI) [24] which is a surrogate for vegetation “vigour” or “greenness”, to more complex indices and analyses depending on whether the image are broadband [25] or hyper-spectral [26]. To determine quantitative information such as biomass requires ground-based calibration of the remotely-sensed image. For example, in temperate regions satellite images are being used to estimate pasture biomass [27] and pasture growth rate [28]. There has been extensive work on using satellite data for calculating net primary productivity [29,30]. However, developing calibration equations for mapping pasture biomass from satellite data in rangeland and savannah environments [31] is complicated due to difficulties in collecting the ground validation data necessary for calibration. In rangeland and savannah environments pastures are highly heterogeneous, with mixed plant species of different phenology, a wide range in biomass and the amount of exposed soil background. The spatial scale of many remotely-sensed images is too coarse to represent this heterogeneity. In tropical environments, the predominance of tall “tussock” grasses makes ground-based measurement of biomass difficult. We focus here on the remote sensing of pastures rather than other landscape features such as trees.

Within herbivore grazing systems, independent information derived from remotely-sensed images is used to infer relationships between the animal's landscape preferences and the inferred vegetation characteristics. These layers of inference introduce uncertainty, which may be reduced by directly correlating herbivore preferences based on GPS monitoring of herbivore movement with their landscape preferences. This approach reduces the uncertainty associated with the inference methods and removes the need to obtain ground-based vegetation calibration data. Wireless sensor networks enable high temporal-frequency GPS monitoring of animal locations to be directly linked to the spatially extensive measurements from remotely-sensed satellite images. An additional advantage of using WSNs is that no direct user involvement is required to download data from the devices, as is the case with traditional data loggers mounted on animals, and the data are streamed to the user in real-time. Studies that have combined multiple sensors within an integrated environment are rare and reflect the technological constraints of integration. Wark and others [8] showed preliminary work on how ground-based multi-spectral sensors and satellite remotely-sensed data may be combined using a WSN. Bro-Jørgensen and others [32] showed how satellite-derived NDVI could be used to explain ranging patterns in antelope behaviour.

Radio-transceivers and passive radio frequency identification (RFID) devices have been used to record information on animal ID and more recently to explore social interactions [33]. In particular, transceivers worn by a pair of animals can collect information on social encounters. The devices, referred to as contact or proximity loggers, record the date, time and duration of a close encounter. The inter-animal distance that is recorded as an encounter by the proximity logger can be adjusted by varying the transmission power setting of the device. Proximity loggers have been used to explore social interactions between cows and calves and also to explore potential risks of disease transmission by recording contacts between wild and domesticated animals [14,34]. The extensive deployment of proximity loggers as part of a WSN provides the opportunity to explore animal social encounters at broad spatial and temporal scales. In addressing these concepts our research interests are motivated by the desire to both identify tools for managing animals and for more sustainable land management.

We recognise that the specific details of cattle behaviour may not be of widespread interest, however, the aim of this paper is to use the work we have undertaken to date to demonstrate some of the broader issues, challenges and opportunities of animal data collected using WSNs integrated with satellite remote sensing. The ideas and discussion presented in this paper are highlighted using examples from data sets collected during experiments conducted at an extensive cattle research station located in northern Australia. These current results demonstrate the potential for mobile WSNs to:

- Monitor behavioural preferences;
- Quantify social behaviour and;
- Integrate data from ground based animal sensors with remote sensing data to understand animal-landscape interactions.

In this paper, we explore these concepts by examining three functional features of WSNs – sensing, communication and integration, and their associated constraints. We demonstrate how a WSN is able to deliver functional outputs for each of these components and in so doing address important animal-based production, ecological and environmental science and management questions. Finally, we note that although the concepts and conclusions in this paper are drawn from experiments involving domesticated cattle, we believe the principles of mobile WSN applications and their integration with remote sensing could be equally applied to the study of wild animal populations.

## 2. Sensing: Using Mobile Sensors to Monitor Animal Behaviour

### 2.1. Locating animals using GPS

Ecologists and environmental scientists have made varied use of radio tracking and GPS to date to provide data to understand the behavioural ecology of free ranging animals [4-7]. High sample-rate monitoring of animals using current telemetry systems is constrained by the amount of battery energy available to run the devices and the processing and analysis time for these large datasets. Recent work using high sample-rate GPS explored the links between prediction error and GPS fix-rate for cattle grazing in extensive pasture systems [35]. The results demonstrated that a GPS location needs to be collected at least once every 10 seconds to be able to predict selection of patches that are 10 m^2^ with at least 90% accuracy. As precision requirements increase, the burden on data storage increases, but as the interval between GPS fixes increases predictive power decreases [35]. The precision relationship is further confounded by the animal's movement, both its speed and direction. In the simplest case where the animal moves either slowly or in a straight line, then the sample interval can increase with no associated loss in predictive ability.

### 2.2. Study areas

**Figure 1.**
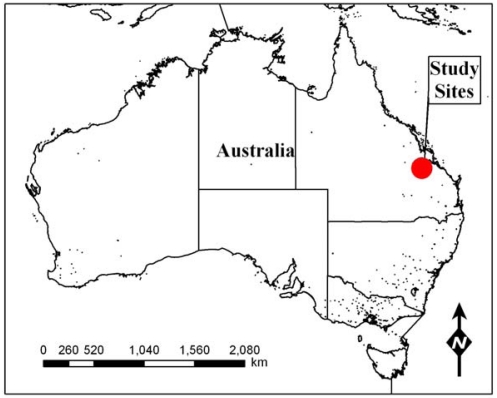
Location of WSN activities at the Belmont Research Station, near Rockhampton (Qld.) Australia, and a nearby study site at Pondicherry. (Spatial data source: Geoscience Australia, 2009)

The majority of the examples used in this paper are selected from WSN research activities conducted at Belmont Research Station, 26 km NNW of Rockhampton (Qld.), in north-eastern Australia (Figure 1) [150° 13′ E, 23° 8′ S]. The research station is located in the Brigalow tropical savannah ecoregion [36], and runs a herd comprised predominantly of Brahman cattle. A second study site was nearby at Pondicherry, 10 km SSW of Dululu (Qld.) [150° 17′ E, 23° 55′ S].

## 3. Communication: Using a WSN to explore Animal Affiliations

### 3.1. Transmission and compression of GPS data

**Figure 2.**
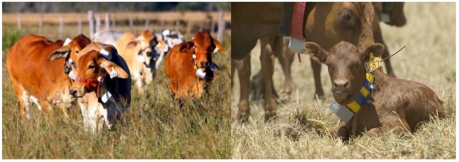
(a) Cattle wearing GPS collars. (b) Cow and calf wearing proximity loggers.

**Figure 3.**
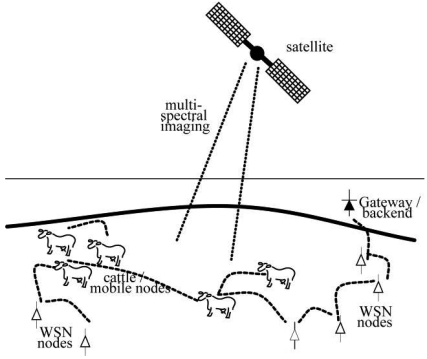
Schematic of mobile animal sensors communicating with each other and an array of static nodes.

As part of the WSN activities animals fitted with GPS collars (Figure 2) can provide positional data at rates of up to 4 Hz (four times per second). These mobile animal sensors are able to communicate with an array of static nodes (Figure 3) to return data to a central base station. However, within this WSN there are bandwidth and energy limitations that affect the amount of data that can be transmitted. For the current system, the bandwidth limit (where nodes are simultaneously trying to communicate over a shared medium) is reached when data are transmitted by five GPS collars at the rate of 4 Hz, given the current 50 kbps radio bandwidth at 915 MHz. Despite this, recent behavioural studies have simultaneously monitored in excess of 40 animals; to successfully transmit these data across the WSN requires data compression methods. Transmission is one of the most power intensive activities for a device, so high data-transfer volumes will more quickly deplete the battery. For both these reasons compression of data prior to transmission is important. To achieve this, data can be selected for transmission based on relevance to the analysis.

**Figure 4.**
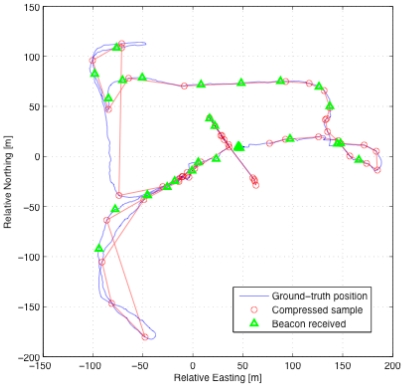
Compression of trajectory data for an animal moving around a paddock. The beacons in the figure are data transmission points.

Furthermore, to enable the collection of data from large herds of cattle a compression algorithm was developed to run on the collar nodes [37] to reduce the amount of data that needs to be transmitted across the network whilst still ensuring accurate trajectory information is returned. Figure 4 shows an example of the complete trajectory data set (stored locally on the device for validation) and compares this with the trajectory derived using compressed data sent over the WSN. By assessing the relative value of individual points, based on the particular analysis goals, data that have low predictive value can be removed. A trajectory can then be determined from the remaining data with relatively high precision within the constraints of network bandwidth and on-board memory resources.

### 3.2. WSN components and deployment

As previously described, WSNs comprise groups of devices (nodes) distributed in the environment which are able to individually measure various parameters (sensors) as well as wirelessly communicate with neighbouring nodes. Collectively the network of nodes provides a means to measure an environmental region at a high temporal resolution and, depending on the availability of sensors, potentially over large spatial extents.

The hardware platform for all nodes in our deployment at Belmont Research Station is based around a sensor platform known as a ‘Fleck’ [9]. The Fleck™ platform uses an Atmega128 micro-controller and a Nordic NRF905 radio transceiver operating in the 915 MHz band. Static nodes are powered from a combination of rechargeable NiMH batteries and solar cells enabling them to run without human intervention. Mobile (cow) nodes also include a GPS and SD card holder daughterboard. For a sensor node to maintain continuous operation under variable weather conditions, the node can be set to run at very low duty cycles where system components such as the radio and/or micro-controller stay asleep for long periods of time to conserve energy.

The Belmont WSN uses a multi-hop topology where data are directed from each node to a single sink point (gateway). Protocols running on each node are used to estimate the quality of radio links, allowing efficient data transmission using the highest quality links. From the gateway, data are streamed to a remote database from where it can be displayed on a web interface in near real-time or input to analysis tools.

## 4. Integration: Sensing the Animal in Its Environment Using a WSN

### 4.1. Proximity loggers and animal social interactions

Animal social interactions are of interest to scientists studying animal behaviour [38-41] since they not only provide information on group dynamics, but also provide valuable information on population dynamics. For example, mapping encounters between males and females can be correlated with mating events, enabling studies of gene flows through a population [42]. Studying interactions between individuals from, within, and between species can be used to map potential disease transmission routes. Proximity loggers are smaller than GPS devices and have lower power requirements enabling them to be deployed for longer periods of time [43]. While proximity loggers are unable to provide direct geo-spatial information, if they are linked to a static sensor network then the proximity of a mobile node to static node with known coordinates enables pseudo-spatial information to be derived. The animal interaction data can be wirelessly transmitted across the WSN, freeing up memory on the device to enable ongoing long-term monitoring studies.

**Figure 5.**
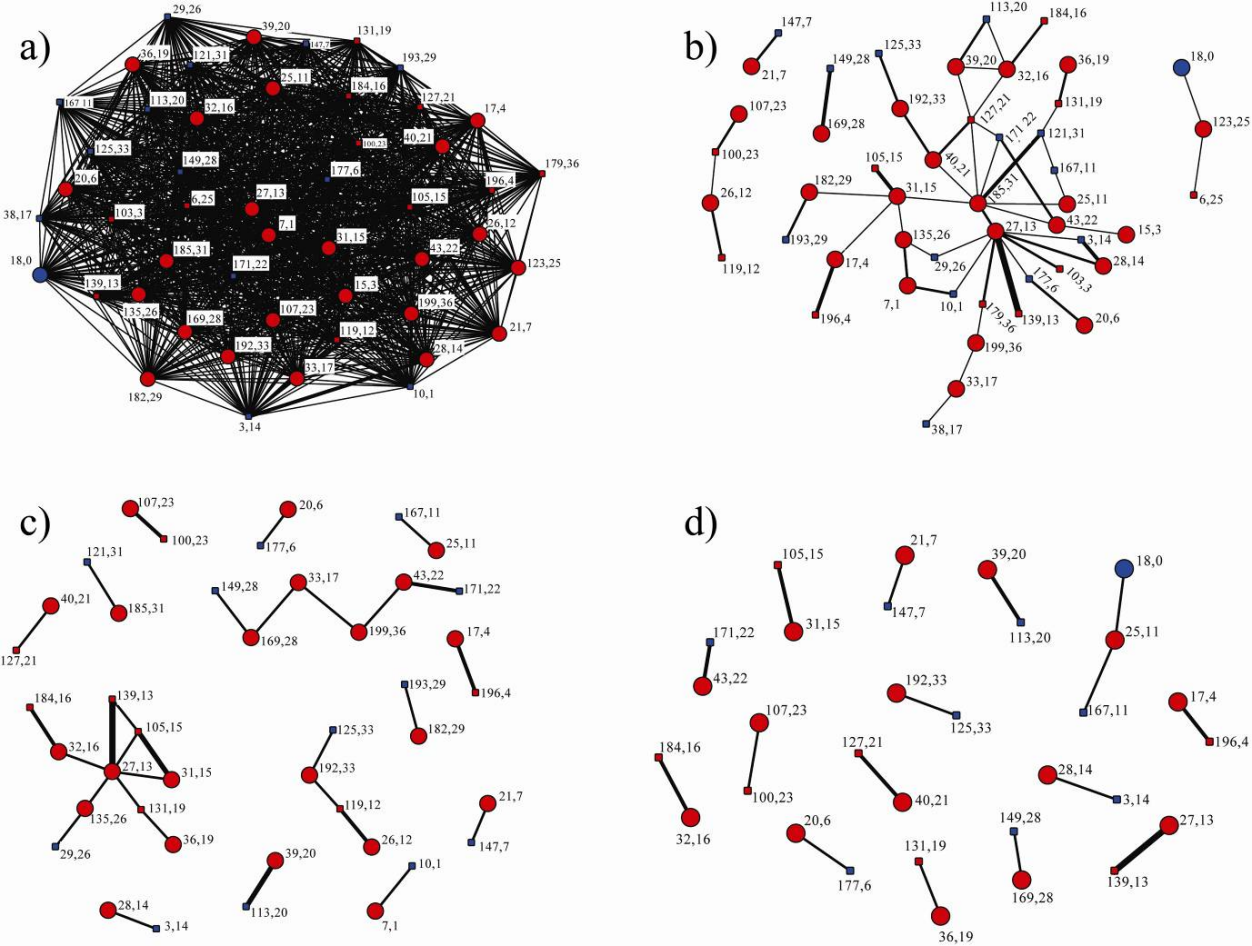
Network diagrams representing total contact duration of contacts less than 5 m between animals logged by proximity loggers. (a) The complete network on day 2, based on 49 animals. (b) The network on day 2 where total contact time was greater than 40 minutes. (c) The network on day 4 where total contact time was greater than 60 minutes. (d) The network on day 6 where contact time was greater than 80 minutes. Isolates (those individuals with no contacts within the specified timeframe) in diagrams (b)-(d) have been removed. Key: colour: red = female, blue = male; shape: circle = adult, square = calf; label: first label is contact logger ID, second label is cow/calf pair code (same pair code equals identified cow/calf pair); thickness of lines represents association strength.

A trial carried out at the Pondicherry study site investigated close (less than 5 m) social interactions of a group of 49 cattle including males, females (sexually active and sexually inactive) and their most recent offspring (Figure 5) and demonstrated preferential social affiliations. The social networks seen in this example display the hierarchy of social interactions and identify key individuals that were more central to the group's social network. The highest level of social affiliation (contact durations of greater than two hours) was between females and their offspring (Figure 5d). However, one cow (number 25) had a strong relationship with a number of individuals in the group. Given the strong relationship between cow number 27 and the bull, it is likely that cow number 27 is in oestrus and is the focus of a sexually active group of females (Figure 5c).

Examples in Figure 5, with different degrees of complexity in the hierarchy, show that by monitoring long-term changes in male and female contacts it is possible to identify social groups and track mating events. The social contacts shown in Figure 5b reveal important sub-groups within the herd. A number of cows (numbers 27, 31 and 185) formed small social sub-groups. Whilst the data of longer duration contacts demonstrates there are preferential relationships within the herd of cattle, the data of short-duration contacts (Figure 5a) shows that there was also interaction between all animals in the group.

### 4.2. Linking animal sensors with remote sensing to understand animal landscape interactions

Integrating telemetry data, in particular GPS data that record animal locations, with remote sensing data allows animal preferences to be directly linked to the spatially extensive measurements of the landscape from airborne- or satellite- images. Once the relationship between animal behaviour and the spectral- and spatial-analysis of the remotely-sensed images is modelled, these relationships can be extended to new images to predict and map likely animal behaviour. Such information can be used to better manage animal movements and protect sensitive areas from degradation through over-grazing.

**Figure 6.**
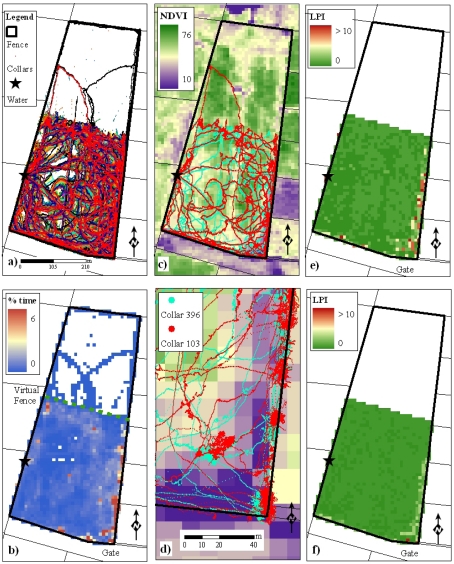
(a) The trajectories of 36 animals over three days in a paddock at Belmont Research Station. (b) Percentage of time during the experiment animals spent in each pixel. (c) Trajectories of two animals overlaid on satellite-derived NDVI values. (d) Same as (c) zoomed into the bottom right corner of the paddock. (e) LPI for each pixel in front of the fence based on the proportion of NDVI in this area for animal number 103. (f) Same as (e) for animal number 396.

Herbivores move around the landscape and preferentially select locations to occupy; these locations will have a set of preferred attributes. As the herbivores interact with their environment, spatial- and temporal-patterns and relationships begin to emerge. We explore how remote sensing might be able to discern behavioural patterns by overlaying high sample-rate GPS data collected from a group of 36 Brahman cattle onto a remotely-sensed image. The cattle were located within a 21 hectare paddock on Belmont Research Station for three days from 3^rd^ April 2008 (Figure 6a). The group of cattle had unlimited access to food and water during the trial. In the examples presented in this paper the animals were kept in one part of the paddock by the use of a virtual fence [8]. Apart from a small number of excursions into the exclusion zone, the animals spent their time on one side of the virtual fence as a “sub paddock”.

A SPOT-5 (10 m pixels) [44] satellite image was acquired on 31^st^ March 2008, and has four spectral bands (green, red, near-infrared (NIR), short-wave infrared). The satellite image was obtained as radiance at sensor from SPOT-Australia, calibrated, processed to top-of-atmosphere reflectance and geo-referenced to base data by Landgate (WA). NDVI was calculated as follows from the red and NIR bands [24]:

(1) NDVI=(NIR−red)/(NIR−red)

The NDVI is sensitive to green biomass rather than dry/dead biomass which is not photosynthetically active. The index ranges from -1 to +1, and NDVIs for green biomass generally fall into the range of approximately 0.3 to 0.8. Higher values of NDVI correlate to relatively “lush” vegetation and lower values of NDVI correlate to lower biomass values, with saturation occurring for high biomass values.

The GPS-derived behavioural data monitored through the WSN was correlated with a number of remote sensing features. Figures 6c-d show the tracks of the animals from GPS collars overlaid with the satellite-derived NDVI and demonstrate a correlation with NDVI. The two animals presented travelled a similar path around the paddock. The animals avoided a “strip” of lower NDVI values in the middle of the paddock; visual assessment of this strip confirmed it contained shorter grass. The animals spent a large amount of time at the south end of the paddock near the gate which is the area with the lowest NDVI values corresponding to lowest forage availability. This behaviour is due to a hierarchy of behavioural drivers where the animal's curiosity for the novelty of the gate and fences overrode the repulsion of the lack of biomass in this area.

**Figure 7.**
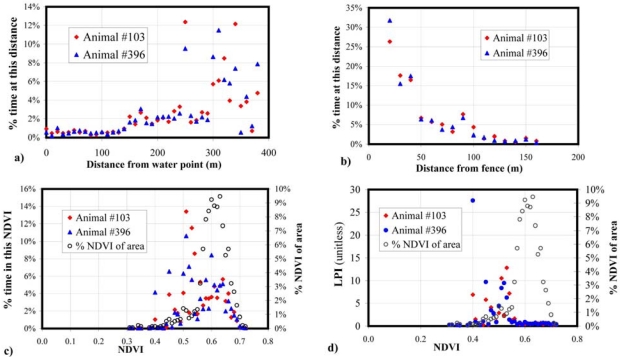
For animals number 103 and 396 during the three day trial, and for the area in front of the virtual fence, (a) the percentage time spent at each distance from the watering point. (b) The percentage time spent at each distance from the fence lines, both virtual and physical. (c) The percentage time spent within pixels of each NDVI value (d) The LPI for each NDVI value.

In this experiment the animals spend the majority of their time around the fences and away from the watering point (Figures 6a-b, 7a-b). This is supported by Figure 7b, which shows the percentage of time that two of the animals spent at different distances from the fence line. For these two animals, the relationship between their behaviour and the time they spent in various parts of the paddock has been further quantified by showing the percentage of time that two animals spent in each NDVI class (Figure 7c). For these two animals the majority of their time is spent at an NDVI of around 0.5, with variation in the distribution of times in pixels with each NDVI, although the most frequent NDVI values are around 0.6.

Whilst raw reflectance values from the remotely-sensed satellite image enable pixels to be grouped according to their specific properties and provides a good starting point to explore correlations, it ignores spatial constraints. For example, the attractiveness of a pixel is in part a feature of clustering; a large group of similar pixels is more obvious and easier to find than a single isolated pixel of the same value. Equally a pixel that is located close to a watering point will have a greater probability of being visited compared to a pixel that is found in a less frequented location. A widely-applied concept that ecologists use to quantify preferential selection of the landscape is to compare the time that an animal spends in a predefined landscape area [45]. We have applied this concept to the present example where the satellite-derived NDVI is used to define the landscape, expressing the method of landscape preference here as the Landscape Preference Index (LPI):

(2) $$\text{LPI}=\frac{\text{proportinal time spent in area of interest}}{\text{proportion of the area of interest compared to whole area available}}$$

Figure 7d shows the LPI associated with NDVI for two animals in the Belmont experiment, with the highest LPI occurring for NDVI values of around 0.5 for animal number 103, and around 0.4 for animal number 396. LPI is calculated only for pixels in front of the virtual fence as the animals had limited access outside this area. As NDVI is a surrogate for vegetation greenness and indicates higher quality feed, these LPI results demonstrate the cattle's preference for greener vegetation, and also the individual variation between animals. The LPI for each NDVI contrasts with the total amount of time animals occupy the different NDVI levels. For example, the low NDVI areas near the gate (Figures 6c-d) have higher LPI values (Figures 6e-f) even though this is a small area of the paddock; this is not unexpected as animals often spend a large amount of time near gates and fences.

These examples show the link between animal behaviour and its environment through the use of GPS collar data and satellite images. Further analyses could explore links with behavioural state, for example when an animal was grazing or resting [4,9] and the correlation with available feed resources, or analysed separately for resting and grazing periods. However, this is work for future research and outside the scope of the present paper. These results highlight that proper interpretation of image and animal movement data requires spectral image information to be combined with spatial analysis and knowledge of animal behaviour.

## 5. Constraints to Integration

### 5.1. Logistical / contextual constraints of sensors within a WSN

Through the examples presented in this paper we have demonstrated some of the issues, challenges and opportunities of using animal-based WSNs. We have seen the advantages of an integrated approach with the combination of sensors, such as GPS location recorders, proximity loggers and satellite-based remote sensing to provide enhanced observation and interpretation opportunities. This integrated approach has allowed animals to be studied in their environment, and allowed greater understanding of animal-landscape interactions which can be linked to management options to achieve more sustainable landscape management. The combination of animal and landscape sensors within the framework of a WSN has a number of spatial, temporal and technological constraints which are summarized in Table 1.

As mentioned previously, two of the key issues with using animal-based sensors are those of storing sufficient energy and data retrieval. This is of particular importance when animals are away from the WSN for long periods of time and battery energy is being expended by broadcasting a signal that is not being received. This necessity to store data until the animals are back in range of the WSN can be mitigated by using the animals themselves as receiving nodes for data and becoming mobile relay nodes rather than having to rely on each animal coming into contact with a static node [9,14]. However, this has an added battery cost to animals whose collars expend extra energy in relaying data.

Other technical and logistical issues to setting up a WSN include the choice and placement of the node infrastructure, determining if the connectivity to nodes is sufficient and spatially covers all parts of the paddock, and physical constraints such as protecting the node structures from being destroyed by animals. The type of sensor being deployed will also influence the infrastructure setup, for example, multi-spectral nodes should not be located under trees where lighting is variable, animal collars must have rugged casings, and animals must be familiarized with wearing collars. Additionally, the mix of sensor types and their spatial arrangement in the WSN needs to encompass the spatial heterogeneity across the farm and the requirements of the experimental design.

### 5.2. Temporal constraints

One of the challenges in combining different sensors is the range of spatial and temporal scales and associated logistical and analysis issues. It is possible to measure animal locations with high frequency, for example 4 Hz as discussed previously within the limits of power and data storage constraints for the system. Remote sensing from air- or space-borne sensors can provide a snapshot of the landscape ranging in frequency from hours or days to months depending on the system, flight or orbit timings, and issues with cost, manpower (for air-borne systems), or cloud-cover (for optical systems). The timing of remote images compared to the observations of animal locations may therefore not match up to the animal behaviour at critical times. This concurrency of data from different sensors is particularly problematic for time-sensitive management of animal behaviour and control of their environmental impact.

Constraints of image timing can be overcome by obtaining more frequent data, for example, the recently launched RapidEye [46] constellation of satellites which aims to provide better than daily coverage of the planet. Recent work using Unmanned Aerial Vehicles (UAVs) to capture remote-sensing data [47] has the potential to give more frequent spatial coverage at lower cost and at temporal frequencies closer to animal- and ground-based sensors. However, these sensors typically come with a number of restrictions; the more frequent satellite-based platforms typically have fewer spectral bands. This limits the information that can be extracted from the images since no matter how fine the pixel size of the data, the sensor must observe the earth at specific spectral wavelengths to allow analysis for each specific application. For example, using short-wave infrared data to observe soils or dry vegetation, NIR data for green vegetation, or thermal infrared data for water and land temperatures. This is critical for observing landscape conditions to match with animal behaviour, as merely having a true-colour “picture” of the landscape may not be suitable for extracting the information necessary for management.

### 5.3. Spatial constraints

The spatial extent of the animal locations being monitored using the WSN and the extent and spatial arrangement of the landscape that is being monitored are also important relative to the pixel size of the image data. For example, depending on the pixel size, a relatively uniform patch of grass will have a different spectral response and texture compared to the patchiness typical in arid rangelands and must be interpreted accordingly. The extent and pixel size of the remotely-sensed images also needs to be matched to the spatial scale of the data from animal-based sensors. For example, although satellite-based sensors such as NASA-MODIS [48] give twice-daily planetary coverage, the 250 to 1,000 m pixel size of the image data is too coarse for interpretation of fine spatial-resolution data from 4 Hz GPS collars. However, for longer-term monitoring of animals such as of cattle between musters, where the recording frequency of ground-based sensors would be much lower, the broad spatial coverage of the NASA-MODIS data would be appropriate. At critical times such as during annual calving, finer-resolution satellite-images covering a smaller extent on the ground could be used and the sample-rate of the animal-based sensors could be increased accordingly.

## 6. Conclusions

We have presented a conceptual overview and discussion around combining ground-based sensing and remote-sensing observations within a WSN to derive improved information about animal behaviour in the environment. Cattle behaviour monitoring data have been used to explore a number of concepts around correlating GPS data and satellite remote-sensing data for improved information about herd behaviour as a function of the animals' environment.

The use of WSNs was proposed as the means for linking these data sources without the need for direct human intervention, allowing a more rapid and efficient means of collecting environmental data than for the individual technologies not linked within a WSN. In animal studies where wired sensor infrastructure is not an option, technological advances in WSNs including on-board processing, miniaturization, and power-use efficiency, are enabling even greater integration of sensor and communication protocols. For example, compression of trajectory data can take place on the nodes worn by the animals The principles of mobile WSN applications we present here using experiments involving domesticated cattle could equally be applied to the study of wild animal populations.

Perhaps the greatest revolution in WSNs is in the integration of sensor systems, where multiple sensor technologies are seamlessly combined with satellite-based remote-sensing data [8]. By combining a diverse array of sensing technologies it is possible to achieve greater understanding of animal-landscape interactions. This was seen in the combination of the GPS data with the remote-sensing data, and in the spatial analysis of the data collected from the contact loggers. More importantly, the implementation of these sensors within the WSN differs from traditional remote monitoring of animals in that data can be collected and transmitted back to the researcher in near real time for analysis and can include data from different sensors, resolutions and sample rates.

Addressing the variation in temporal-sampling frequencies and spatial resolution in each sensing modality remains an open and active research topic; however, the advancing state of WSNs and remote-sensing technologies undoubtedly increases the importance and opportunities in these research areas. Given the current issues around climate change and environmentally sustainability, improving our ability to observe and understand our natural environment will be critical for sustainable land management.

## Figures and Tables

**Table 1.** Constraints to an integrated system of ground-, animal- and satellite-based sensors within a WSN framework.

	**Spatial constraints**	**Temporal constraints**	**Logistical / contextual constraints of sensors within a WSN**
**Ground-based sensors within a WSN**	Sensor node can only observe phenomena in immediate vicinity Distance between nodes is bounded in order to achieve radio communication.	Energy availability (solar or battery) and communications bandwidth which determines the radio duty cycle and maximum data throughput.	Maintenance of sensors and the WSN over long periods Changes in sensor viewing-geometry due to factors such as changes in the distance between the ground/vegetation and the sensor as the vegetation grows. Other viewing-environment effects such as shadows and sun angles. Destruction of sensors or infrastructure by animals.
**Animal-based sensors**	Distance of sensor to the static WSN (communications) or availability of nearby animals as “relay nodes”	Energy availability (battery) and communications bandwidth determines the maximum data throughput	Battery life v/s frequency of measurement Logistics of attaching sensors on animals Maintenance of sensors over long periods, including malfunctions. Size of datasets and associated processing times. Destruction of sensors by animals.
**Satellite remote-sensing**	Pixel size of data v/s extent of animal data Extent of images covering footprint of animal movement	Availability of satellite-images suitable to animal behaviour being studied, such as the grazing preference. Acquisition frequency (daily, monthly, annual) restricted by sensor orbit and cloud cover. Timing of images (morning v/s afternoon overpasses) Data access in time to make management decisions	Sensor having suitable wavelengths to observe the landscape under study Calibration to quantitative values requires seasonal calibration data Limited operational life-span of satellite-based sensors (years to decades)
